# Gamma-Ray-Induced Amino Acid Formation during Aqueous Alteration in Small Bodies: The Effects of Compositions of Starting Solutions

**DOI:** 10.3390/life14010103

**Published:** 2024-01-09

**Authors:** Akari Ishikawa, Yoko Kebukawa, Kensei Kobayashi, Isao Yoda

**Affiliations:** 1 Department of Chemistry and Life Science, Yokohama National University, 79-5 Tokiwadai, Hodogaya-ku, Yokohama 240-8501, Japankobayashi-kensei-wv@ynu.ac.jp (K.K.); 2 Department of Earth and Planetary Sciences, Tokyo Institute of Technology, 2-12-1 Ookayama, Meguro-ku, Tokyo 152-8551, Japan; 3 Co60 Irradiation Facility, Laboratory for Zero-Carbon Energy, Institute of Innovative Research, Tokyo Institute of Technology, 2-12-1 Ookayama, Meguro-ku, Tokyo 152-8550, Japan

**Keywords:** aqueous alteration, carbonaceous chondrites, amino acids, prebiotic chemistry

## Abstract

Organic compounds, such as amino acids, are essential for the origin of life, and they may have been delivered to the prebiotic Earth from extra-terrestrial sources, such as carbonaceous chondrites. In the parent bodies of carbonaceous chondrites, the radioactive decays of short-lived radionuclides, such as ^26^Al, cause the melting of ice, and aqueous alteration occurs in the early stages of solar system formation. Many experimental studies have shown that complex organic matter, including amino acids and high-molecular-weight organic compounds, is produced by such hydrothermal processes. On the other hand, radiation, particularly gamma rays from radionuclides, can contribute to the formation of amino acids from simple molecules such as formaldehyde and ammonia. In this study, we investigated the details of gamma-ray-induced amino acid formation, focusing on the effects of different starting materials on aqueous solutions of formaldehyde, ammonia, methanol, and glycolaldehyde with various compositions, as well as hexamethylenetetramine. Alanine and glycine were the most abundantly formed amino acids after acid hydrolysis of gamma-ray-irradiated products. Amino acid formation increased with increasing gamma-ray irradiation doses. Lower amounts of ammonia relative to formaldehyde produced more amino acids. Glycolaldehyde significantly increased amino acid yields. Our results indicated that glycolaldehyde formation from formaldehyde enhanced by gamma rays is key for the subsequent production of amino acids.

## 1. Introduction

Amino acids (AAs) are essential molecules for living organisms on Earth. Proteins consist of AAs, and free AAs are responsible for diverse functions such as signal transduction and endocrine tissue regulation. The synthesis of AAs on prebiotic earth has been hypothesized in various ways. AAs are believed to be chemically synthesized from methane, ammonia, hydrogen, and water using the energy of lightning and geothermal heat, and peptides and proteins are produced through chemical evolution, e.g., [1,2,3]. Prebiotic pathways other than that pioneered by Miller and Urey [1] are those obtained from the homologation of hydrogen cyanide in the presence of sulfides [4] and the reductive amination of simple alpha-keto acids in the presence of iron powder [5]. Because indigenous AAs have been found in carbonaceous chondrites [6], they are thought to have been delivered to the prebiotic Earth. Currently, a total of 95 amino acids have been identified in the Murchison meteorite [7]. AAs also have been detected in various meteorites, including thermally metamorphosed chondrites and primitive ureilites, e.g., [8,9]. The wide diversity in the abundance and distribution of amino acids in various chondrites indicates that the parent body process largely affects the AA composition, in addition to the pre-alteration materials present in parent bodies [10].

AAs and their precursors can be synthesized on icy grains in interstellar molecular clouds by photochemistry followed by warming up in the protosolar nebula [11,12,13,14,15,16,17]. In addition, catalytic gas-phase reactions such as Fischer–Tropsch type (FTT) synthesis in high-temperature environments in protosolar nebula or parent asteroids can produce AAs from H_2_, CO, and NH_3_ [8,18,19,20]. Alternatively, AAs can be synthesized from aldehydes, cyanides, ketones, and ammonia via hydrothermal processes inside the parent bodies of carbonaceous chondrites, e.g., [10,21].

Recent studies have shown that hydrothermal ”formose-type” reactions—formose reaction is the formation of sugars from formaldehyde—starting from aldehydes and ammonia or hexamethylenetetramine (HMT, C_6_H_12_N_4_), which easily decompose into formaldehyde and ammonia, produce amino acids [7,22,23,24,25,26]. The most plausible heat sources for the hydrothermal reaction were short-lived radioactive nuclides such as ^26^Al which was abundant in the parent bodies at the early stage of the Solar System [27]; thus, gamma rays from these nuclides would have contributed to the reactions. In contrast to hydrothermal reactions, gamma-ray irradiation of aqueous solutions of formaldehyde, ammonia, and methanol produces various AAs, the yields of which depend on the total dose of gamma-ray irradiation [28]. However, the effects of the starting composition on the formation of AAs are unknown. Therefore, in this study, we conducted gamma-ray irradiation experiments to investigate the effects of the composition of the starting aqueous solutions on AA formation: (1) the ratios of ammonia, (2) the presence of glycolaldehyde (GA), and (3) the presence of HMT as a precursor of formaldehyde and ammonia, including the effects of gamma-ray dose rates and comparisons with heating experiments.

## 2. Materials and Methods

### 2.1. Gamma-Ray Irradiation Experiments

Four groups of starting aqueous solutions were prepared for gamma-ray irradiation experiments: (1) ammonia, formaldehyde, and methanol with the molar ratios of H_2_O: NH_3_: HCHO:CH_3_OH = 100:0–10:0–10:0–1.66; (2) ammonia, formaldehyde, glycolaldehyde, and methanol with molar ratios of H_2_O:NH_3_:HCHO:C_2_H_4_O_2_:CH_3_OH = 100:(1 or 5):5:1:0.83; (3) ammonia, formaldehyde, glycolaldehyde, methanol, and calcium hydroxide with the molar ratios of H_2_O:NH_3_:HCHO:C_2_H_4_O_2_:CH_3_OH:Ca(OH)_2_ = 100:0.54:3.6:1.8:0.6:0.36 (based on Kebukawa et al. 2013 [29]), and (4) hexamethylenetetramine (HMT) with the molar ratios of H_2_O: HMT = 100:1. These sets of the starting compositions were selected to investigate (1) the effects of the ratios of ammonia; (2) the effects of glycolaldehyde which is the initial compounds of formose reaction and the formation of glycolaldehyde is kinetically slow without catalysts such as Ca(OH)_2_ and/or glycolaldehyde (self-catalyst); (3) comparison to the previous amino acid formation experiments by heating [22]; and (4) to see whether HMT is indeed an alternative source of formaldehyde and ammonia since HMT is easily decomposed to these compounds through: HMT + 6 H_2_O ⇋ 6 HCHO + 4 NH_3_ [23]. However, the decomposition of HMT takes time; the half-life of hydrolysis of HMT at 30 °C is estimated to be 160 days at pH 7 [30]. Therefore, we also examined the effect of HMT decomposition by leaving HMT aqueous solutions at room temperature for 168 h (one week) before the gamma-ray irradiation experiments. Ammonia, formaldehyde, and methanol are abundant in comets—the initial composition of the parent bodies of organic-rich chondrites prior to aqueous alteration could be similar to comets—as H_2_O:NH_3_:HCHO:CH_3_OH = 100:≤1.5:≤4:≤4 [31]. Glycolaldehyde has been found in comets but not as abundant as formaldehyde 0.02% relative to water in C/2014 Q2 (Lovejoy) [32], and 0.4% relative to water in 67P/Churyumov–Gerasimenko [33]. HMT is not found in comets so far, but abundantly produced by experimental studies simulating icy dust particles [34,35,36], and also found in chondritic meteorites [37].

The abbreviations used for the starting aqueous solutions are shown in Table 1 and include the letters “A” for ammonia, “F” for formaldehyde, “G” for glycolaldehyde, “W” for water, and “Ca” for calcium hydroxide. For the FAW samples, if the ratio of HCHO to 100 water is 5, it is written as FAW(*x*) (*x* is the ratio of NH_3_); if the ratio is a value other than 5, it is written as FAW(*x*/*y*) (*y* is the ratio of HCHO); and FGAWCa and HMT are presented without numbers because we used fixed ratios for these samples. The sample HMT(rt) was left at room temperature for 168 h after sample preparation to allow time for HMT to decompose into HCHO and NH_3_, and then gamma-ray irradiation/heating experiments were conducted.

Aqueous solutions were prepared by mixing the reagents to match the molar ratios shown in Table 1. For example, FAW(5) samples were prepared in vials using 0.340 g of 25% (2.5 M) NH_3_ aqueous solution (FUJIFILM Wako Pure Chemical, Osaka, Japan), 0.405 g of 37% (2.5 M) HCHO aqueous solution (containing 5% methanol, FUJIFILM Wako Pure Chemical), and 1.29 g of pure water obtained from Milli-Q system (Merck Millipore, Burlington, MA, USA) to prepare H_2_O:NH_3_:HCHO:CH_3_OH = 100:5:5:0.84 mixtures. The FGAW(5) samples were prepared in vials using 0.340 g of 25% NH_3_ aqueous solution, 0.405 g of 37% HCHO aqueous solution, 0.06 g of glycolaldehyde dimer powder (Sigma-Aldrich, St. Louis, MO, USA), and 1.29 g of pure water to make H_2_O:NH_3_:HCHO:C_2_H_4_O_2_:CH_3_OH = 100:5:5:1:0.84 mixtures. The FGAWCa samples were prepared in a vial using 0.0368 g of 25% NH_3_ aqueous solution, 0.291 g of 37% HCHO aqueous solution, 0.108 g of glycolaldehyde dimer powder, 0.0267 g of calcium hydroxide powder (FUJIFILM Wako Pure Chemical), and 1.75 g of pure water to make H_2_O:NH_3_:HCHO:C_2_H_4_O_2_:CH_3_OH:Ca(OH)_2_ = 100:0.54:3.6:1.8:0.6:0.36 mixtures. The HMT samples were prepared in a vial using 0.140 g of hexamethylenetetramine powder (Wako) and 1.801 g of pure water to make HMT:H_2_O = 1:100 mixtures. A 200 μL aliquot of each solution was placed in a glass tube (6 mm in diameter) using a micropipette. The glass tubes were placed in liquid nitrogen to freeze the aqueous solution, and the tubes were flame sealed while being evacuated to remove oxygen, which might have affected the reactions.

The sealed glass tubes were irradiated by gamma rays at room temperature at various dose rates (0.15 to 1.5 kGy/h) and durations (5 to 600 h) using a ^60^Co gamma-ray source at the Zero Carbon Research Institute, Institute for the Creation of Science and Technology, Tokyo Institute of Technology. To ensure the effects of gamma rays, control samples were prepared in the same way and analyzed immediately after preparation of the aqueous solutions or after leaving the samples at room temperature for 600 h after preparation. We also prepared FAW(0) (without NH_3_) and FAW(5/0) (without HCHO and CH_3_OH) as the procedural blanks. In addition, heating experiments (without gamma rays) were performed for comparison purposes. Because the maximum temperatures of aqueous alteration were estimated to be up to 80 °C for CM2 chondrites and 150 °C for CI chondrites [27], heating experiments were conducted at 150 °C for 24, 48, or 72 h or at 80 °C for 162 h in an oven (ETTAS HTO-450s) for selected starting solutions. The experimental conditions are listed in Table 1. Duplicate experiments were conducted by preparing two sample tubes with identical starting solutions followed by gamma-ray irradiation with the same conditions, for FAW(5), (10), (5/10), (5/0), and HMT(rt) with gamma-ray irradiation at 1.5 and 0.15 kGy/h for 60 and 600 h, and reasonable reproducibility was confirmed.

### 2.2. Amino Acid Analysis

AA analysis was performed as described by Kebukawa et al. (2022) [28]. The samples were subjected to acid hydrolysis at 110 °C by aluminum block thermostatic chambers (SIBATA DBH-1000, ADVANTEC TPB-32) for 24 h with 5 M HCl (1 mL of 6 M HCl diluted from 12 M hydrochloric acid (FUJIFILM Wako Pure Chemical) was added to 200 μL of sample) to liberate bound AAs, a common technique for AA analysis. After acid hydrolysis, samples were dried via vacuum centrifugation (EYELA CVE-3110) at 60 °C. The samples were dissolved in 1 mL Milli-Q water. Each resulting aqueous solution was filtered through membrane filters (DISMIC-25CS, ADVANTEC) and analyzed using a high-performance liquid chromatography (HPLC) system. The HPLC system consisted of a system controller (Shimadzu CBM-20A), an HPLC pump (Shimadzu LC-20AD), a polystyrene ion column heated to 60 °C with a column heater (Shimadzu CTO-20AD) (Shimadzu Shimpack ISC-07/S1504), and a fluorescence detector (Shimadzu RF-20Axs) with an excitation wavelength of 358 nm and emission wavelength of 450 nm. The post-column derivatization method was performed using a solute consisting of 0.65 g/L O-phthalaldehyde, 0.104 g/L N-acetyl-L-cysteine, 40.7 g/L sodium carbonate, 13.5 g/L boric acid, 18.8 g/L potassium sulfate, and 0.8 g/L ethanol. All reagents used for derivatization were purchased from FUJIFILM Wako Pure Chemicals. Gradient elution was performed using an amino acid mobile phase kit (Na type, Shimadzu) with the following solutions: (A) pH 3.23 sodium citrate buffer (containing 0.2 M Na^+^ and 7% (*v*/*v*) ethanol); (B) pH 10.00 sodium citrate buffer (containing approximately 0.73 M Na^+^ and 0.2 M boric acid); and (C) 0.2 M sodium hydroxide solution (for conditioning). The flow rate of the carrier solution was 0.300 mL min^–1^ with (A) and (B) gradients; 100% (A) at first 0–15 min, the ratio of (B) was increased from 0% to 16% at 15–35 min, 16% (B) was kept at 35–40 min, the ratio of (B) was increased from 60% to 100% at 40–50 min, 100% (B) at 50–60 min, and, finally, 100% (A) at 60–65 min. Commercially available AA standard solutions (AA Mixed Standard Solution AN-II type and B type, FUJIFILM Wako Pure Chemical) were used for the quantitative analysis of glycine (Gly), alanine (Ala), β-alanine (β-Ala), serine (Ser), α-aminobutyric acid (α-ABA), β-aminoisobutyric acid (β-AIB), γ-aminobutyric acid (γ-ABA), threonine (Thr), aspartic acid (Asp), valine (Val), glutamic acid (Glu), isoleucine (Ile), leucine (Leu), and α-aminoadipic acid (α-AAA). The AAs were identified based on the retention time using standards, and the possibility of the formation of other AAs cannot be ruled out. The detection limits of AAs (except α-ABA and α-AAA) were approximately 0.05 μM, and those of α-ABA and α-AAA were approximately 0.5 μM.

## 3. Results

### 3.1. Formation of AAs by Gamma-Ray Irradiation

**Figure 1:**
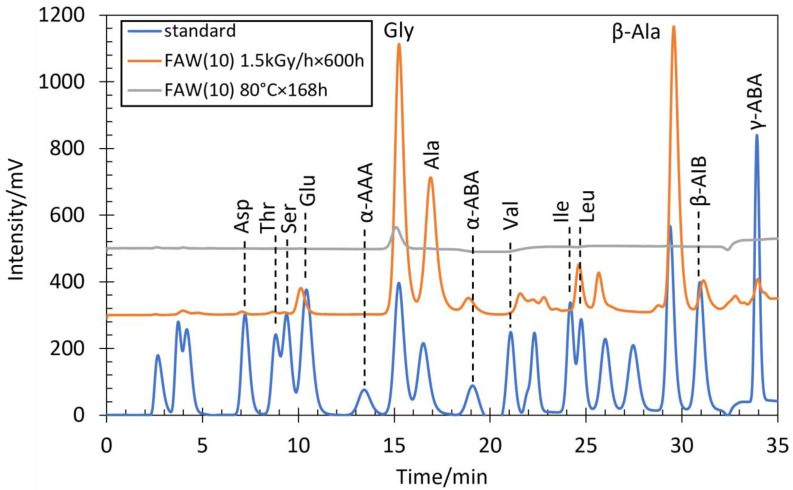
HPLC chromatograms of FAW(10) after gamma-ray irradiation at 1.5 kGy/h × 600 h, FAW(10) after heating at 80 °C for 168 h after acid hydrolysis, and AA standard solution.

Several AAs were identified by HPLC analysis in all gamma-ray irradiated samples after acid hydrolysis as well as in the heated samples after acid hydrolysis. The AA concentrations detected in all samples analyzed by HPLC are shown in Table S1. Figure 1 shows chromatograms of representative FAW(10) sample after gamma-ray irradiation at 1.5 kGy/h × 600 h, and FAW(10) sample after heated at 80 °C for 168 h, as well as chromatograms of the AA standard solution obtained from HPLC. The results of the gamma-ray irradiation experiment showed that Ala and Gly were the main products, and some β-Ala, Ser, α-ABA, β-AIB, γ-ABA, Thr, Asp, Val, Glu, Ile, Leu, and α-AAA were present as well. Duplicate experiments were conducted under the selected conditions, and reasonable reproducibility was confirmed. The uncertainties were likely due to experimental handling and uncertainty of gamma-ray irradiation conditions. Such uncertainties were expected to be much larger than the analytical uncertainty, which is not discussed here. In addition, we prepared procedural blanks without C or N sources [FAW(0) and FAW(5/0)]. The AAs detected from these blanks were up to 2.7 μM in total, and mostly consisted of Gly, indicating that contamination from the experimental and analytical procedures was less than ~3 μM.

We also prepared control samples without gamma-ray irradiation for each starting composition to confirm the effects of the gamma rays. Most of the FAW and HMT samples produced more AAs by gamma-ray irradiation than the controls with the same starting solutions, except under the following conditions: 9 kGy (0.15 kGy/h × 60), 90 kGy (0.15 kGy/h × 600 h), 90 kGy (1.5 kGy/h × 60 h) of FAW(5), and 7.5 kGy (1.5 kGy/h × 5 h) of HMT. In 84% of the FAW samples (26 out of 31 samples) and 91% of the HMT samples (10 out of 11 samples), more AAs were produced by gamma-ray irradiation than in the control. On the other hand, most of the gamma-ray irradiated FGAW samples produced comparable or fewer AAs than the controls (see Effects of GA Section).

Almost no AAs were detected (0.1 μM in total) in the gamma-ray-irradiated (25 kGy) FAW(5) without acid hydrolysis, whereas small amounts of Ala and β-Ala (73 μM and 34 μM, respectively) were detected in the heated (150 °C, 72 h) FAW(5) without acid hydrolysis (Table S1). This indicates that gamma-ray irradiation of the starting solution produces AA precursors. The AA precursors were likely to be in the form of higher-molecular-weight compounds that release AAs by acid hydrolysis. Simple Ala and β-Ala were also formed in the free state (detected without acid hydrolysis) in heated FAW, and acid hydrolysis increased the amounts of AAs produced. This indicates that both free AAs and AA precursors were present in the heated sample. Furthermore, the free AAs were relatively simple Ala and β-Ala; however, acid hydrolysis of the AA precursors resulted in the detection of many high-molecular-weight AAs.

**Figure 2:**
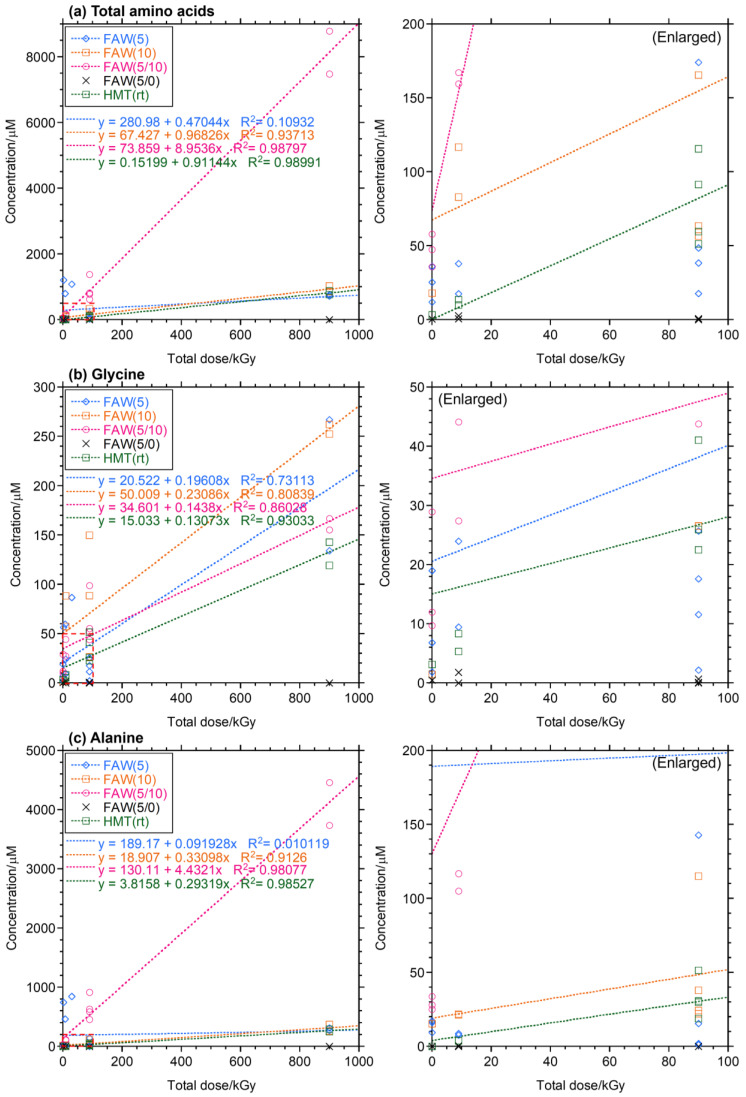
Yields of (**a**) total AAs, (**b**) Gly, and (**c**) Ala with gamma-ray doses of the gamma-ray irradiated samples. Right: enlarged to show the ranges shown in dotted squares.

The yields and types of AAs produced increased with increasing gamma-ray radiation dose and also varied depending on the compositions of the starting solution mixtures. Figure 2 shows the concentrations of total AAs and Ala with the dose of gamma-ray irradiation at various dose rates (0.15, 0.5, and 1.5 kGy/h). The concentrations of AAs increased linearly (*R*^2^ = 0.81–0.99) with the gamma-ray irradiation dose in FAW(5/10), FAW(10), and HMT(rt), but the AA yields from FAW(5) did not correlate with their total gamma-ray doses. FAW(5/10) samples showed a particularly marked increase, which may be due to the higher amount of HCHO added compared to these samples, i.e., their higher carbon content.

### 3.2. Effects of the Starting Solutions for the Formation of AAs by Gamma-Ray Irradiation

**Figure 3:**
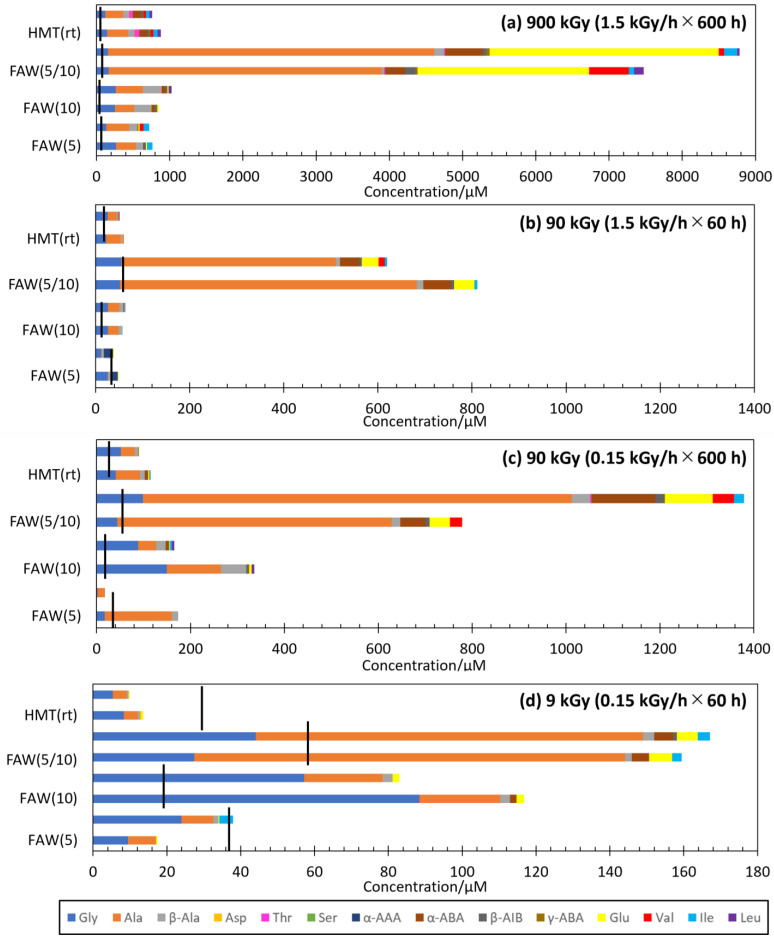
Results of AA formation for each gamma-ray radiation dose: (**a**) 1.5 kGy/h × 600 h = 900 kGy, (**b**) 1.5 kGy/h × 60 h = 90 kGy, (**c**) 0.15 kGy/h × 600 h = 90 kGy, and (**d**) 0.15 kGy/h × 60 h = 9 kGy. Black lines indicate the AA concentrations in the control samples. The largest values are shown if there are multiple control samples (the largest values from either 0 h or 600 h) from the same starting solutions.

The types and yields of AAs depend largely on the starting solutions. Figure 3 compares the AA yields from FAW(5), FAW(10), FAW(5/10), and HMT(rt) by gamma-ray irradiation at 900 kGy (1.5 kGy/h × 600 h), 90 kGy (1.5 kGy/h × 60 h), 90 kGy (0.15 kGy/h × 600 h), and 9 kGy (0.15 kGy/h × 60 h). The FAW (5/10) samples produced the largest amounts of AAs and the largest variations in AAs under all irradiation conditions (Figure 3). In particular, 900 kGy (1.5 kGy/h × 600 h) of gamma rays produced one order of magnitude larger concentrations of AAs in FAW(5/10) than those in FAW(5), FAW(10), and HMT(rt). The types of AAs in the FAW(5/10) showed larger variations. In FAW(5) and FAW(10), Glu and α-ABA were detected in 1–5% and 2% of all amino acids, respectively, while in FAW(5/10), Glu and α-ABA were detected in 6–22% and 25–35%, respectively (Figure 3a). Since Glu and α-ABA have higher C numbers than Gly and Ala, the reactions probably progressed more in FAW(5/10). Also, an increase in glutamic acid at higher total gamma-ray dose was also observed in Kebukawa et al. (2022) [28].

AA yields are known to linearly increase with the total gamma-ray dose, independent of dose rates [28]. Therefore, it can be expected that the AA yields would be similar under conditions of 1.5 kGy/h × 60 h and 0.15 kGy/h × 600 h (both 90 kGy in total). Indeed, the AA yields from the 1.5 kGy/h × 60 h irradiated samples were similar to those from the 0.15 kGy/h × 600 h irradiated samples in samples FAW(5), FAW(10), FAW(5/10), and HMT(rt) (Figure 3b,c). We confirmed that the total gamma-ray irradiation dose had a strong influence on AA production at various starting compositions. However, slightly more AAs were produced in the 0.15 kGy/h × 600 h irradiated samples compared to those samples that used the same starting solutions (Figure 3b,c). Therefore, longer reaction durations and lower dose rates would be more efficient for AA formation by gamma rays.

### 3.3. Effects of the HCHO/NH_3_ Ratios in the Starting Solutions on the Formation of AAs by Gamma-Ray Irradiation

**Figure 4:**
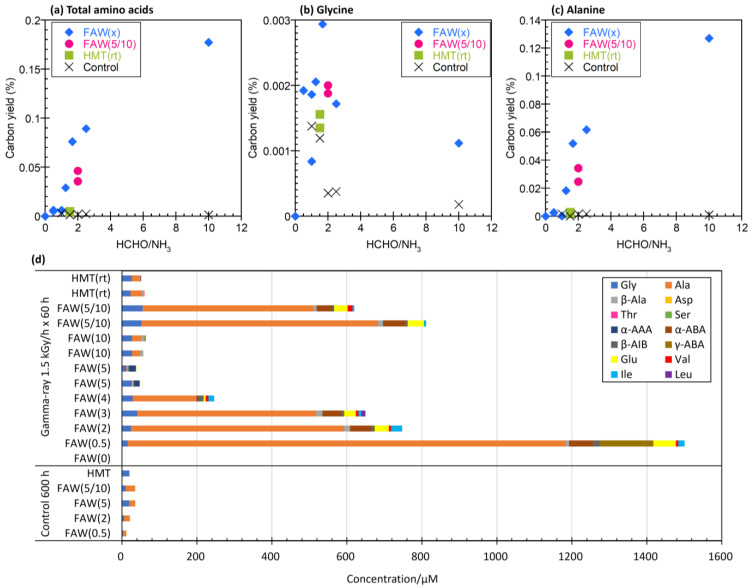
Carbon yield (%) of (**a**) total AAs, (**b**) Gly, and (**c**) Ala by gamma-ray irradiation at 90 kGy (1.5 kGy/h × 60 h) and control samples (600 h at room temperature) with the HCHO/NH_3_ ratios of the starting solutions. (**d**) Each AA yields from the samples shown in (**a**–**c**) and 600 h control samples of the same starting solution.

To evaluate the effects of the ratio of HCHO to NH_3_ in the starting solutions, the carbon yield—the total moles of C in AAs produced by gamma-ray irradiation divided by the total moles of C in the starting solution—was plotted against the HCHO/NH_3_ ratios (Figure 4a). Because the concentrations of HCHO in the starting solutions varied, the AA yields were compared with the ratios of C converted from HCHO in the starting solutions. For HMT (C_6_H_12_N_4_), the HCHO/NH_3_ ratio was considered to be equivalent to 6/4 = 1.5. Figure 4a shows that the AA yields increased with increasing HCHO/NH_3_ ratios. In particular, a large difference was observed between FAW(2) (HCHO/NH_3_ = 2.5) and FAW(0.5) (HCHO/NH_3_ = 10). Figure 4b shows the yield of each AA from the samples shown in Figure 4a. The AAs produced by gamma-ray irradiation were mainly Ala, and the Ala yields increased when NH_3_ was lower in the starting solutions.

### 3.4. HMT as a Starting Material Instead of HCHO and NH_3_

**Figure 5:**
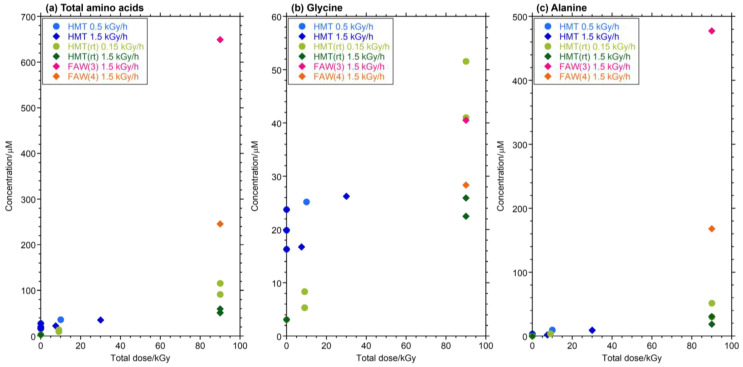
Yields of (**a**) total AAs, (**b**) Gly, and (**c**) Ala from HMT by gamma-ray irradiation and control experiments.

HMT was used as the starting material instead of HCHO and NH_3_. HMT is known to produce AAs via hydrothermal reactions, probably via decomposition into HCHO and NH_3_ [23,38]. In the FAW sample, HCHO and NH_3_ were present in the starting solution from the beginning, but in the HMT sample, they were supplied over time, so we assumed that there was a change in the way AAs were formed in the FAW and HMT samples. Therefore, the experiment was conducted under the assumption that there would be a change in the way AAs were formed between FAW and HMT. HMT(rt) samples irradiated after leaving them at room temperature for 168 h were compared to HMT samples irradiated immediately after preparation. Note that the HMT samples were kept in the freezer overnight before gamma-ray irradiation, and the samples were outside the freezer for transportation for approximately 3–4 h. Figure 5 shows AAs produced from HMT(rt) by gamma-ray irradiation at 90 kGy (1.5 kGy/h × 60 h and 0.15 kGy/h × 600 h) and 9 kGy (0.15 kGy/h × 60 h), HMT (without leaving at room temperature) with gamma-ray irradiation at 7.5 kGy (1.5 kGy/h × 5 h), 30 kGy (1.5 kGy/h × 20 h), and 10 kGy (0.5k Gy/h × 20 h), and FAW(3) and FAW(4) (similar C/N ratios with HMT) with gamma-ray irradiation at 90 kGy (1.5 kGy/h × 60 h) are shown for comparison. The AA yields from HMT and HMT (rt) increased with the total irradiation dose (Figure 5). No obvious increase in AAs was observed when the samples were left at room temperature prior to gamma-ray irradiation of the HTM, although a simple comparison of HMT and HMT(rt) is difficult owing to the differences in the irradiation conditions (Figure 5). However, when FAW(3), FAW(4), and HMT were compared, the concentrations of AAs produced by FAW(3) and FAW(4) were larger than those produced by HMT(rt) at a gamma-ray dose of 90 kGy. This indicates that FAW more efficiently produces AAs than HMT, probably because the amounts of HCHO and NH_3_ released from HMT are low at room temperature.

### 3.5. Effects of Glycolaldehyde (GA) and Calcium Hydroxyde in the Starting Solutions on the Formation of AAs by Gamma-Ray Irradiation

**Figure 6:**
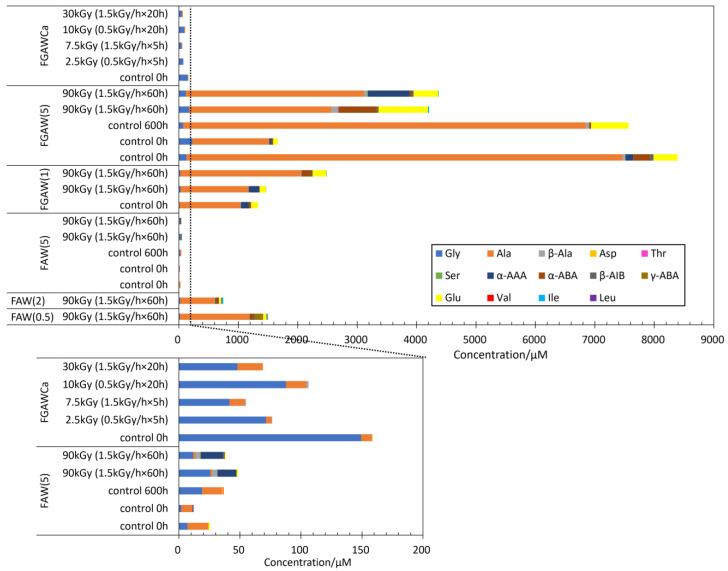
AA yields from the gamma-ray irradiation experiments from FGAWCa, FGAW(1), and FGAW (5) compared to FAW(5), FAW(2), and (FAW(0.5).

Figure 6 shows the yields of AAs from FGAW and FGAWCa. The AA yields from all FGAW and FGAWCa samples after gamma-ray irradiation were comparable to the AA yields from the corresponding controls. While AAs produced by the heating of FGAWCa at 150 °C for 48 h and 72 h were higher than the control, 24 h of heating produced a comparable amount of AAs to the control. In the FGAW(5) samples, longer chain α-amino acids including α-ABA, Glu, and α-AAA were preferentially produced. It is probably one of the effects of glycolaldehyde which enhances the formation of longer side chains of amino acids.

Overall, the AA yields from FGAWCa were significantly lower than those from FGAW(5) and FGAW(1). Since Ca(OH)_2_ is an effective catalyst for the formation reaction and enhances the production of macromolecular organic matter [39,40], reactions other than those related to the formation of amino acids may have been more advanced in the FGAWCa samples.

### 3.6. Differences in AA Formations in Gamma-Ray Irradiation and Heating Experiments

**Figure 7:**
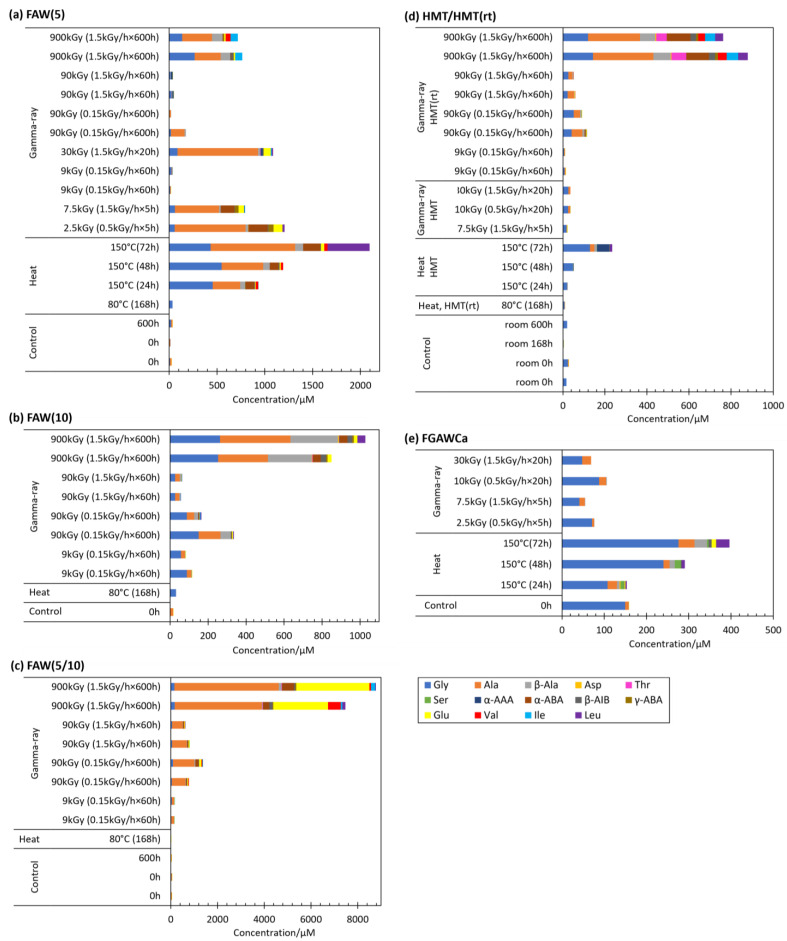
AA yields from the gamma-ray irradiation experiments compared to the results of the heating experiments and controls. (**a**) FAW(5), (**b**) FAW(10), (**c**) FAW(5/10), (**d**) HMT/HMT(rt), and (**e**) FGAWCa.

Heating experiments were conducted for selected starting solutions at 80 °C or 150 °C to compare the effects of temperature. The AA yields from the gamma-ray irradiation, heating, and control experiments are shown in Figure 7 for each starting solution. In many cases, AA yields from the heating experiments were larger than those of controls, except for the yields from the 80 °C and 168 h heating experiments, and the 150 °C and 24 h heating experiments of HMT and FGAWCa (Figure 7). In the case of FAW(5), AA formations were in the following order (from largest to smallest): 150 °C heating > 900 kGy of gamma ray > 80 °C heating. In HMT/HMT(rt), the order was 900 kGy of gamma ray > 150 °C heating > 80 °C heating. The total AA yields from FAW(5), FAW(10), FAW(5/10), and HMT at 80 °C heating were as low as those of the control samples. The gamma-ray irradiation and heating results for FAW(5) and HMT showed that samples heated to 150 °C produced more AAs than gamma-ray irradiation, while heating to 80 °C produced fewer AAs than gamma-ray irradiation, 150 °C heating produced more AAs than 80 °C heating, and 150 °C heating was more effective in AA production; as the heating time increased, the amounts of AAs produced also increased at 150 °C. When 150 °C heating was performed, the reaction of AA production did not reach equilibrium, and further AA production would be expected if heating was continued. On the other hand, heating at 80 °C produced almost no AAs. The reason for the small amounts of AA produced may be because of the low temperature, even though the heating time was 168 h, which is long enough for the AA formation reaction to reach equilibrium.

**Figure 8:**
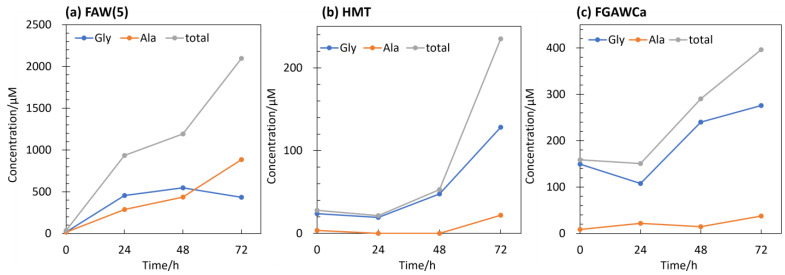
Gly, Ala, and total AAs yields from samples heated at 150 °C for 24, 48, and 72 h and controls (0 h) of (**a**) FAW(5), (**b**) HMT, and (**c**) FGAWCa.

There was also a difference in the type of AAs produced: at 80 °C heating, Gly was the dominant AA produced, with small amounts of other AAs such as Ala, β-Ala, and Asp. Figure 8 shows the amounts of Gly, Ala, and total AAs produced by the FAW(5), FGAWCa, and HMT samples heated at 150 °C for 24, 48, and 72 h. When heated at 150 °C for up to 72 h, Ala was detected in large amounts (22–885 μM) from solutions, and Leu, α-ABA, and β-Ala also showed increased yields (Figure 7a). There was a clear difference between the 48 and 72 h samples, suggesting that the formation of higher-carbon Aas progressed between 48 h and 72 h. Comparing the results of these heating/control experiments with those of gamma-ray irradiation, greater amounts of Aas were produced by gamma-ray irradiation from most of the starting solutions, although this result depended on the intensity of the gamma-ray radiation. The types of Aas produced were mostly Gly in the heated samples, Ala and other Aas were produced in several of the heated and control samples, while a wide variety of Aas were produced in abundance under gamma-ray irradiation.

## 4. Discussion

### 4.1. Effects of Gamma Rays on the Formation of AAs from Aqueous Formose-Type Reaction

Although the initial compositions of chondrite parent bodies before aqueous alteration are unknown, one can expect that simple molecules such as NH_3_, HCHO, and CH_3_OH are abundant because comets that experienced limited aqueous activities contain these compounds up to an NH_3_:HCHO:CH_3_OH:H_2_O = 1.5:4:4:100 in molar ratio [31]. Aldehydes and ammonia are known to produce various organic compounds, including amino acids [7,22,24,25,26,41], sugars [39,42], alkyl pyridines, N-heterocyclic compounds [43,44], and various soluble and insoluble organic compounds [25,29,40,45,46,47,48]. In addition, hexamethylenetetramine (HMT, C_6_H_12_N_4_) is an alternative starting material to NH_3_ and HCHO. HMT is a major product of laboratory experiments that simulate the photochemistry of icy dust particles [34,35,36,49,50,51,52,53,54]. HMT is known to produce various organic compounds, including amino acids, after hydrothermal reactions simulate parent body aqueous alteration at 150 °C, probably via decomposition of HMT into NH_3_ and HCHO [23,38,55]. NH_3_, HCHO, and HMT have been found in carbonaceous chondrites; for example, the Murchison meteorite was found to contain 1100 nmol/g of NH_3_ [56], 67 nmol/g of HCHO [57], and 6 nmol/g (846 ppb) of HMT [37]. It is reasonable that these concentrations are much smaller than those of the comets, although HMT was not detected in comets, since these substances are highly reactive and are mostly consumed by reactions occurring during aqueous alteration in parent bodies. The major heat source for aqueous alteration is considered to be the decay of radioactive nuclides, such as ^26^Al [58,59,60], although other possibilities such as impact heating, e.g., [61], and gas-phase hydration of silicates in the nebula, e.g., [62], cannot be excluded. The excess ^26^Mg in chondrites indicates that ^26^Al was abundant in parent bodies at the early stage of Solar System formation [59,60]. The β^+^ decay of the ^26^Al nuclide produces 3.12 MeV per atom mostly as gamma rays [63]. In the case of the CM chondrite, we previously estimated that the total gamma-ray irradiation produced in the parent body was approximately 6.3 MGy [28], based on the canonical values for the ^26^Al/^27^Al ratio (~5 × 10^–5^) [60] and the abundance of Al in the Murchison meteorite (1.14 wt %) [64]. Although the half-life of ^26^Al (7.17 × 10^5^ y) is “short” relative to the timescale of the Solar System, it is too long for us to use ^26^Al as a gamma-ray source; thus, we use the ^60^Co (the half-life of 5.27 y) gamma-ray source instead of ^26^Al in our experiments.

Gamma-ray irradiation of aqueous solutions containing NH_3_, HCHO, and CH_3_OH produced abundant AAs, mainly Ala, Gly, β-Ala, α-ABA, γ-ABA, β-AIB, Glu, Val, Ile, and Leu after acid hydrolysis. In most cases, the AA yields showed roughly linear relationships with the total gamma-ray irradiation dose, independent of the dose rates (Figure 2). This is consistent with previous gamma-ray irradiation experiments [28], even when the dose rate (up to 20 kGy/h) was higher than that used in the present experiment (up to 1.5 kGy/h).

Acid hydrolysis is commonly performed for amino acid analysis from chondrites to release AAs that are bound to larger compounds, e.g., [65]. Acid hydrolysis was conducted before AA analysis, as in our previous studies [22,24,28], and related studies from other groups [7,26]. We performed AA analysis without acid hydrolysis, but almost no detectable AAs were obtained by gamma-ray irradiation, and small amounts of AAs (~5% of the hydrolyzed sample) were detected after the heating experiments. These results are consistent with our previous gamma-ray irradiation experiment [28], and heating experiments (~25% of the hydrolyzed samples [22]). In general, higher abundances of “free” amino acids (detected without acid hydrolysis) were produced from heating experiments than from gamma-ray irradiation. Most of the AAs obtained after gamma-ray irradiation and heating were in the form of AA precursors. The molecular structures of AA precursors are not well known, but a candidate precursor of Ala is alanineamide [7], or compounds with higher molecular weights containing amide bonds that reveal AAs by acid hydrolysis.

We found that GA formation is key to the production of AAs. With the addition of GA to the starting solutions, even the samples without gamma rays or heating (control samples) produced considerable amounts of AAs (Figure 6). GA is the initial product of the formation of sugars from HCHO via formose reaction. The initial process is kinetically slow; however, once GA is formed, the formose reaction proceeds through self-catalytic reactions [66]. On the other hand, it is known that GA was produced from HCHO by gamma-ray irradiations [67]. Thus, FAW samples exposed to gamma-ray irradiation might initially form GA, and then a formose-type reaction involving NH_3_ can produce AA precursors. Ala formation was previously considered to occur via the formation of acetaldehyde from ethylene glycol via gamma-ray radiolysis [28,68]. However, our results indicate that acetaldehyde can be produced from GA instead of ethylene glycol by gamma-ray radiolysis [69] or from reduction of GA [70]. The rest of the reaction proceeds as proposed by Kebukawa et al. (2022) [28]: acetaldehyde reacts with ammonia to produce ethanimine [71], which then produces CH_3_CHNH_2_ radicals and reacts with NH_2_CO radicals to produce alaninamide, which produces alanine via acid hydrolysis [7]. It is noteworthy that Ala was dominant in the AAs from FAGW samples in both the gamma-ray irradiated and control samples, indicating that the reaction mechanism was similar to that of the gamma-ray irradiation of the FAW samples, which resulted in dominant Ala yields. In addition, GA could be used to accelerate the reaction to reach the limit of the reaction in future work; thus far, the AA yields increase with total gamma-ray dose, and the limit of AA formation is unknown.

With respect to the HCHO/NH_3_ ratios in the starting solutions, larger amounts of AAs were produced when the HCHO/NH_3_ ratios (or C/N ratios) were higher, such as, HCHO/NH_3_ molar ratios of 10 [FAW(0.5)] and 2.5 [FAW(2)]. This tendency could be related to the C/N ratio of the AA products. Such high C/N ratios are similar to the C/N ratios of the AAs; the C/N molar ratio of Gly (C_2_H_5_NO_2_) is 2 and the C/N molar ratio of Ala (C_3_H_7_NO_2_) is 3. Ala yields significantly increased with increasing HCHO/NH_3_ ratios (Figure 4). It seems reasonable that higher C/N ratios in the starting solutions produced AAs with higher C/N ratios.

**Figure 9:**
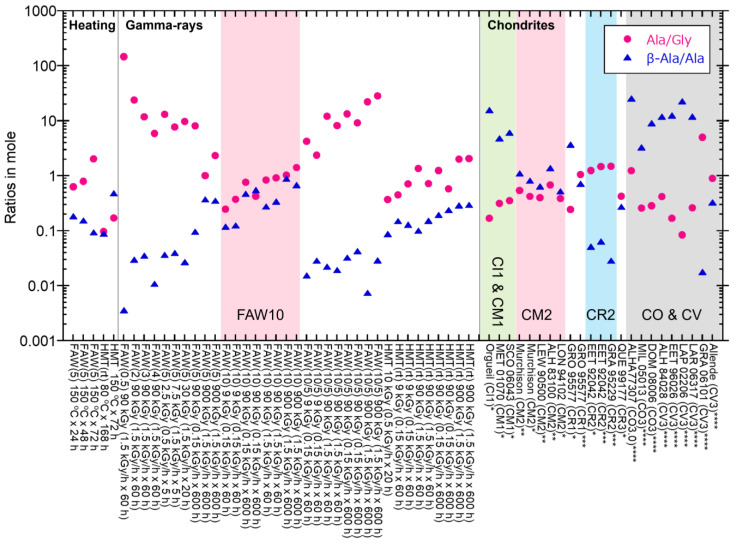
The Ala/Gly and β-Ala/Ala ratios of AAs from the FAW and HMT samples compared to the literature values of carbonaceous chondrites. Data from * Glavin et al. (2011) [73], ** Glavin et al. (2006) [72], *** Martins et al. (2007) [74], and **** Burton et al. (2012) [8].

We also conducted heating experiments at 80 °C and 150 °C for comparison. These temperatures were selected because the alteration temperatures of CM chondrites and CI chondrites were up to 80 °C and 150 °C, respectively [27]. Remarkable differences between gamma rays and hearting are shown in the Ala/Gly ratios (Figure 9); most of the heated samples have Ala/Gly ratios (in moles) of less than 1, while most of the gamma-ray irradiated samples have Ala/Gly ratios larger than 1. These results suggest that the reaction pathways for AA formation differ between gamma-ray irradiation and heating. In heating experiments, Koga and Naraoka (2022) [26] reported AA formations from glycolaldehyde and NH_3_ by heating at 60 °C for 6 days and showed that larger AAs were produced under air atmosphere conditions relative to those produced under N_2_ purge. They proposed that the key reaction is the oxidation of glycolaldehyde, which produces glycinate, and that the combination of two glycinate acids with NH_3_ produces N-oxalylglycine, followed by the formation of Gly via dehydration. In our experiments, the effect of O_2_ was not clear because we eliminated O_2_ from our samples under vacuum by freezing them with liquid nitrogen. However, we cannot exclude the possibility that the remaining O_2_ supports AA formation in our experiments. The AA products in Koga and Naraoka (2022) [26] were dominant in Gly, followed by Ala and β-Ala, which is consistent with our heating experiments but inconsistent with gamma-ray irradiation. This also supports the differences in the reaction mechanisms of AAs formed by heating and gamma rays.

### 4.2. Comparison with Previous Experiments

Compared to our previous experiments that used similar conditions and gamma-ray irradiation of H_2_O:NH_3_:HCHO:CH_3_OH = 100:6:8:1 solutions [28], the AA yields were roughly consistent with the FAW(5/10) (H_2_O:NH_3_:HCHO:CH_3_OH = 100:5:10:1.7) results in this study (Table 2). The 9 kGy gamma-ray irradiations produced 27–44 μM of Gly, 105–117 μM of Ala, and 2–3 μM of β-Ala in this study and 7.5 kGy of irradiations produced 16–84 μM of Gly, 33–64 μM of Ala, and 0.6–2.3 μM of β-Ala in Kebukawa et al. (2022) [28]. The 90 kGy gamma-ray irradiations produced 44–99 μM Gly, 455–913 μM Ala, and 9–39 μM β-Ala in this study, and 100 kGy irradiations produced 66–71 μM Gly, 382–411 μM Ala, and 42–48 μM β-Ala in Kebukawa et al. (2022) [28].

In contrast, heating experiments at 150 °C for 72 h for FGAWCa produced 126 μM of Gly, 29 μM of Ala, and 29 μM of β-Ala (after subtracting AA yields of the control), which was particularly less Ala and β-Ala than that produced in similar experiments in Kebukawa et al. (2017) [22]—278–344 μM of Gly, 479–1081 μM of Ala, and 171–219 μM of β-Ala. FAW (5) (H_2_O:NH_3_:HCHO:CH_3_OH = 100:5:5:0.83) heated at 150 °C for 72 h produced 414 μM of Gly, 867 μM of Ala, and 83 μM of β-Ala (after subtracting AA yields of the control). These values are one or two orders of magnitude larger than those produced by similar experiments of H_2_O:NH_3_:HCHO = 100:1:9 (with some MeOH) in Elmasry et al. (2021) [24]—3.4 μM of Gly, 4.3 μM of Ala, and 5.9 μM of β-Ala. These differences were likely due to differences in experimental and analytical protocols, including the preparation of starting solutions, conditions of acid hydrolysis, and analytical procedures. In particular, the AA analysis by Elmasry et al. (2021) [24] was conducted by reversed-phase HPLC, which probably affected the results compared to other studies that used cation-exchange HPLC.

In this study, heating to 150 °C for 72 h of HMT (HMT:H_2_O = 1:100 equivalent to ~0.6 M) produced 128 μM of Gly, 22 μM of Ala, and 11 μM of β-Ala after acid hydrolysis, while Vinogradoff et al. (2020) [23] reported that heating to 150 °C for 48 h of 0.7 M HMT in alkaline conditions (pH 10) with KOH produced 8 μM of Gly, <0.1 μM of Ala, and 1 μM of β-Ala, without acid hydrolysis. These differences are likely due to acid hydrolysis, but the effect of pH is worth considering in future studies since we did not attempt to control the pH in our experiments.

### 4.3. Comparison to AAs in Chondrites

Since the AAs detected from carbonaceous chondrites have large variations, gamma rays contributed to AA formation inside the parent bodies. Considering that the total gamma-ray dose for CM parent bodies is estimated as ~6.3 MGy, the expected total AA yield is up to 56 mM, calculated by linear extrapolation of AA yields with the total gamma-ray dose of FAW(5/10); [Total AAs]/μM = 8.95 × 6300 (kGy) + 73.9 (Figure 2a). This value indicates that ~4% of the C in the starting solutions was converted to AAs. Specific to Ala, the expected Ala yield was up to 28 mM by 6.3 MGy of gamma rays calculated as [Ala]/μM = 4.43 × 6300 (kGy) + 130 (Figure 2c), which is equivalent to ~2% of C in the starting solutions. The remaining HCHO and NH_3_ are used to produce other compounds. The total Ala production by gamma rays expected in parent bodies can be calculated as follows: 89.9 g/mol × 0.163 × (28 × 10^3^) μM = 410 × 10^3^ ng/g ~ 400 μg/g using a water weight fraction of 0.163 [28]. This value is similar to that obtained in our previous calculation [28]. Note that this is the upper limit of AA yields in parent bodies, and decompositions and other factors affect accentual AA yields. Thus, in the following paragraphs, we discuss the relative abundance of AAs in carbonaceous chondrites and our experimental products.

Compared to the AAs in CM2 chondrites, the Ala/Gly ratios and the β-Ala/Ala ratios in CM2 are 0.39–0.69 and 0.52–1.1 in moles, respectively [72,73], while the Ala/Gly ratios of most of the samples produced by gamma rays in our experiments were much larger, up to 147, but the β-Ala/Ala ratios were similar or lower (up to 0.88) (Figure 9). However, gamma-ray irradiated FAW(10) samples have Ala/Gly ratios of 0.25–1.42, and β-Ala/Ala ratios of 0.12–0.88, which are somewhat similar to the CM2 values (pink shaded in Figure 9). In the case of CI1 and CM1 chondrites (green shaded in Figure 9), the Ala/Gly ratios (0.17–0.35) are lower than Murchison, and the β-Ala/Ala ratios (4.8–15.7) are higher than Murchison [73] and the gamma-ray irradiated samples. The CR2 chondrites (blue shaded in Figure 9) have higher Ala/Gly ratios (1.3–1.5) and lower β-Ala/Ala ratios (0.03–0.06) than those of the CM2 and CI1chondrites [73,74], and these ratios are closer to the AA abundances in gamma-ray irradiated FAW samples. Although these ratios of the most primitive CR3 chondrite are different than those listed above (Ala/Gly ratio of 0.43 and β-Ala/Ala ratio of 0.27), CR2 chondrites are more primitive than CM2 chondrites considering that organic matter in CR2 has higher D (^2^H) and ^15^N abundance [75]. Thus, our results imply that the initial AA compositions in the parent bodies are somewhat similar to those in our gamma-ray irradiated products, and alterations could reduce the Ala/Gly ratios and increase the β-Ala/Ala ratios. This is consistent with the fact that Ala decreases more rapidly than Gly in aqueous solutions [76] and β-Ala is thermodynamically more stable than Ala [77]. This interpretation is also supported by the observation that thermally metamorphosed carbonaceous chondrites (COs and CVs) generally have higher β-Ala/Ala ratios (up to 23) [8] (gray shaded in Figure 9). However, one cannot ignore the effects of the starting compositions, i.e., gamma-ray-irradiated FAW(10) samples have similar Ala/Gly and β-Ala/Ala ratios relative to those of CM2 chondrites. In summary, the AA compositions of meteorites could be due to initial abundances of NH_3_ and HCHO in parent bodies and alteration degrees after AA formation, probably owing to selective decomposition of AAs, e.g., [76,78].

It is interesting to note that high radical abundance was observed in insoluble organic matter (IOM) in carbonaceous chondrites [79,80,81,82,83,84]. These radicals are generally attributed to the ionization environment at the protoplanetary disk before accretion of the parent bodies [82,83,85]. In addition to such environments, gamma rays from the decay of radioactive nuclides in the parent bodies might also cause such radicals in the IOM.

## 5. Conclusions

AAs analysis of gamma-irradiated aqueous solutions of formaldehyde, ammonia, and methanol revealed that Ala and Gly were the most abundant types, followed by β-Ala, α-ABA, γ-ABA, β-AIB, α-AAA, Glu, Val, Leu, Ile, Asp, Ser, and Thr. The AA yields increased with the total gamma-ray dose, as shown in previous experiments [28]. In our experiments, we found the following results:

1. The yields of AAs increased as the HCHO/NH_3_ ratios in the starting solutions increased, indicating that more amino acids were formed when the C/N ratios in the starting solutions were closer to those of amino acids.

2. When HMT, which partially decomposes into formaldehyde and ammonia, was used in the starting solutions, AA formation was not as efficient as that in the HCHO and NH_3_ solutions. This is probably because the amounts of HCHO and NH_3_ released from HMT were low at room temperature.

3. The addition of GA to the starting solution significantly increased amino acid production even in the control experiments. GA is the initial product of the formose reaction and this step is kinetically slow. GA in the starting solutions compensated for this step, suggesting that the formation of GA by gamma rays enhances the formation of AAs from starting solutions without GA.

4. Comparing gamma-ray irradiation and heating experiments, gamma-ray irradiation is more effective for AA formation than 80 °C heating, but comparable to the results of 150 °C heating. The AAs produced by the heating experiment were mostly Gly (except in a few cases), indicating that gamma-ray irradiation and heating likely induce different reaction pathways for AA formation.

5. The Ala/Gly and β-Ala/Ala ratios from the gamma-ray irradiated products were somewhat similar to those from primitive CR2 chondrites (higher Ala/Gly ratios and lower β-Ala/Ala ratios). However, the samples with higher HCHO contents—FAW(10)—show ratios similar to the AAs from CM2 chondrites.

6. A simple extrapolation of Ala yields to the expected total gamma-ray dose indicating that up to ~400 μg/g Ala can be produced in CM parent bodies during aqueous alteration. This value is equivalent to ~2% of the carbon in the starting solution, which can be used for Ala formation.

## Figures and Tables

**Table 1:** Experimental conditions for gamma-ray irradiations, heating experiments, and controls.

	Starting Solutions (Molar Ratio to H_2_O = 100)						Gamma-Ray Irradiation									Heating				Control	
	HCHO	C_2_H_4_O_2_	NH_3_	CH_3_OH	HMT	Ca(OH)_2_	0.15 kGy/h		0.5 kGy/h			1.5 kGy/h				80 °C	150 °C			Room Temperature	
Abbreviations	(F)	(G)	(A)			(Ca)	60 h	600 h	5 h	20 h	50h	5 h	20 h	60 h	600 h	168 h	24 h	48 h	72 h	0 h	600 h
FAW(5/0)	0		5				✔✔	✔✔						✔	✔✔	✔				✔	
FAW(0)	5		0	0.83										✔							
FAW(0.5)	5		0.5	0.83										✔						✔	✔
FAW(2)	5		2	0.83										✔						✔	✔
FAW(3)	5		3	0.83										✔							
FAW(4)	5		4	0.83										✔							
FAW(5)	5		5	0.83			✔✔	✔✔	✔		*	✔	✔	✔✔	✔✔	✔	✔	✔	✔*	✔✔	✔
FAW(10)	5		10	0.83			✔✔	✔✔						✔✔	✔✔	✔				✔	
FAW(5/10)	10		5	1.66			✔✔	✔✔						✔✔	✔✔	✔				✔✔	✔
FGAW(1)	5	1	1	0.83										✔✔						✔	
FGAW(5)	5	1	5	0.83										✔✔						✔✔	✔
FGAWCa	3.6	1.8	0.54	0.6		0.36			✔	✔		✔	✔				✔	✔	✔	✔	
HMT					1					✔		✔	✔				✔	✔	✔	✔✔	✔
HMT(rt)					1		✔✔	✔✔						✔✔	✔✔	✔				**	

The molar ratios of the starting solutions were H_2_O = 100. ✔✔: duplicate experiments. *: without acid hydrolysis. **: leaving at room temperature for 168 h.

**Table 2:** Comparison of amino acid yields to previous studies.

Starting Solution		Condition	Amino Acid Yield (μM)			Reference
			Gly	Ala	β-Ala	
H_2_O:NH_3_:HCHO:CH_3_OH = 100:5:10:1.7	FAW(5/10)	Gamma-ray 9 kGy	27–44	105–117	2–3	This study
H_2_O:NH_3_:HCHO:CH_3_OH = 100:6:8:1		Gamma-ray 7.5 kGy	16–84	33–64	0.6–2.3	Kebukawa et al., 2022 [28]
H_2_O:NH_3_:HCHO:CH_3_OH = 100:5:10:1.7	FAW(5/10)	Gamma-ray 90 kGy	44–99	455–913	9–39	This study
H_2_O:NH_3_:HCHO:CH_3_OH = 100:6:8:1		Gamma-ray 100 kGy	66–71	382–411	42–48	Kebukawa et al., 2022 [28]
H_2_O:NH_3_:HCHO:C_2_H_4_O_2_:CH_3_OH:Ca(OH)_2_ = 100:0.54:3.6:1.8:0.6:0.36	FGAWCa	150 °C for 72 h	126	29	29	This study
H_2_O:NH_3_:HCHO:C_2_H_4_O_2_:Ca(OH)_2_ = 100:0.54:3.6:1.8:0.36		150 °C for 72 h	278–344	479–1081	171–219	Kebukawa et al., 2017 [22]
H_2_O:NH_3_:HCHO:CH_3_OH = 100:5:5:0.83	FAW (5)	150 °C for 72 h	414	867	83	This study (control subtracted)
H_2_O:NH_3_:HCHO = 100:1:9 (with some MeOH)		150 °C for 72 h	3.4	4.3	5.9	Elmasry et al., 2021 [24]
HMT:H_2_O = 1:100 equivalent to ~0.6 M HMT		150 °C for 72 h	128	22	11	This study
0.7 M HMT in alkaline conditions (pH 10) with KOH		150 °C for 48 h (without acid hydrolysis)	8	<0.1	1	Vinogradoff et al., 2020 [23]

## Data Availability

The data that support the findings of this study are available from the corresponding author upon reasonable request.

## Supplementary Materials

The following supporting information can be downloaded at: https://www.mdpi.com/article/10.3390/life14010103/s1. Table S1. Amino acids yields from all gamma-ray irradiated, heated, and control samples. Provided as an Excel sheet.
